# Evolution of the conformational ensemble and allosteric networks of apoptotic caspases in chordates

**DOI:** 10.1042/BCJ20250001

**Published:** 2025-08-05

**Authors:** Isha Joglekar, Mithun Nag Karadi Giridhar, David A. Diaz, Ankit Deo, A. Clay Clark

**Affiliations:** 1 Department of Biology, University of Texas at Arlington, Arlington, Texas 76019, U.S.A.; 2 Department of Information Systems, University of Texas at Arlington, Arlington, Texas 76019, U.S.A.

**Keywords:** caspase, conformational dynamics, energy landscape, folding, network analysis, protein evolution

## Abstract

Apoptotic caspases exist not as static structures but as dynamic ensembles in solution, finely tuned by post-translational modifications and oligomerization. The fine-tuning of this ensemble by cellular cues allows caspases to influence not only apoptotic pathways but also the non-apoptotic pathways in which they are involved. These ensembles span a complex conformational landscape from well-characterized low-energy states captured in structural databases to transient high-energy intermediates that remain elusive and poorly understood. This limited structural view poses a major barrier to fully understanding how caspase activity is regulated and diversified across cellular contexts. To address this, we integrate evolutionary, folding, and mutational data with molecular dynamics simulations and network analysis to uncover a highly conserved residue network in structural space that has been faithfully passed on in sequence space over 500 million years of vertebrate evolution. This network encodes a high-energy intermediate consistently present in the ensemble of all present-day vertebrate apoptotic caspases. It not only guides folding but also scaffolds dynamic motions, functioning like a structural backbone that supports the ensemble. Building on this foundation, we identify differentially evolving networks surrounding the conserved core in initiator and effector caspase subfamilies. These variations provide thermodynamic insight into how initiators stabilize monomeric conformations while effectors favor dimeric states, revealing how evolution shapes ensembles to diversify function in protein families. Additionally, we discover conserved hub residues near an allosteric hotspot, distinct from the core network, that regulate the dynamics of surrounding evolving networks and act as control centers that modulate the conformational equilibrium within the apoptotic caspase ensemble.

## Introduction

Understanding how sequence variation in protein families leads to functional diversity and specificity while conserving the fold is a fundamental challenge in molecular evolution [1,2]. Versatile functionalities in protein families arise from a complex and poorly understood choreography of conformational dynamics [3]. With the surge of data from sequence, structure, biochemical, and biophysical studies, there is a need to integrate these diverse sources and understand their relationships to elucidate how protein families evolve amino acid interaction networks that drive dynamics while preserving the fold [4,5]. Such efforts are important not only for advancing our understanding of molecular evolution but also for developing more precise models of protein dynamics to inform pharmacological strategies by enhancing our understanding of the structure–function relationships in proteins [6].

**Figure 1:**
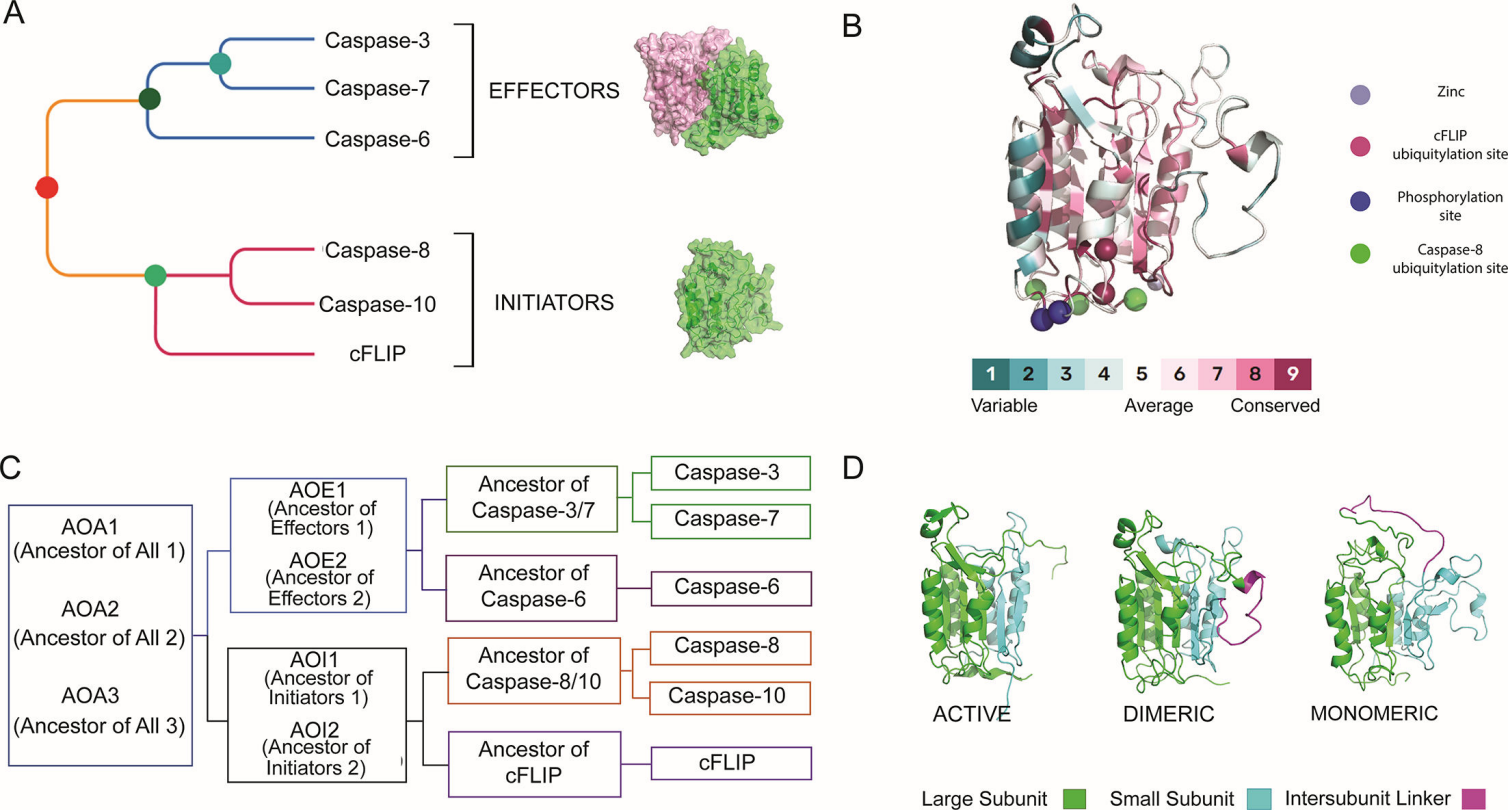
Caspase structure and evolution. (**A**) Evolutionary relationship of caspases involved in the extrinsic pathway of apoptosis. (**B**) Conservation of site-specific residue mapped on caspase-8 (6*P*x9), along with post-translational modification sites and zinc-binding site (depicted as spheres). The legend below indicates the conservation score scale, and the sphere colors on the right represent different types of post-translational modifications. (**C**) Phylogenetic tree illustrating the evolutionary relationships among modern caspases and inferred ancestral nodes. (**D**) Structural representation of caspases in the active, dimeric, and monomeric state, highlighting differences in their conformation, with the large subunit in green, the small subunit in cyan, and the inter-subunit linker in magenta.

Apoptosis is a regulated form of cell death in which a cell orchestrates its own dismantling through specialized molecular machinery. Central to this process are caspases, a family of cysteine-dependent, aspartate-directed proteases that act as key drivers of the apoptotic program [7,8]. Apoptotic caspases have evolved into initiators, manifesting as stable monomers, and effectors, existing as stable dimers (Figure 1A). Their activation mechanisms are paradoxically disparate [9]. Dimerization stands as a pivotal prerequisite for caspase activation, with initiator caspases tightly controlled through orchestrated dimerization within oligomerization platforms in response to apoptotic signals [7,8,10]. Subsequent activation of initiator caspases triggers downstream activation of dimeric effector caspases, thus unraveling an intricate activation mechanism within the apoptotic cascade [11]. However, the phenomenon of dimerization is a multifaceted process that has not been thoroughly understood in either initiator or effector caspases [7,12,13]. Interestingly, folding studies on effector caspases reveal that dimerization is not simply the association of two-folded monomers but rather a key folding event in which partially folded monomeric intermediates interact and co-fold into the dimeric state [14]. Furthermore, while dimerization increases the free energy of effector caspases, the monomeric conformation has conserved free energy in both initiator and effector caspases, suggesting its persistent presence with similar thermodynamic properties in the conformational ensemble of both subfamilies [15,16]. Kinetic folding studies of procaspase-3 support this idea, revealing both dimerization-competent and dimerization-incompetent monomeric species [17]. These findings suggest that initiator caspases have evolved an energetic barrier that stabilizes the monomer as the native state, requiring assistance from dimerizing platforms for activation, whereas effectors remain in equilibrium between monomeric and dimeric states within their conformational ensemble. The apoptotic caspase family, with its well-characterized folding landscape and wealth of structural data, provides a powerful system to explore how conformational ensembles are shaped by the evolution of amino acid networks and folding landscapes within protein families.

In addition to sequence variations that influence conformational ensembles, post-translational modifications also alter the equilibrium between states, modulating caspase function [7]. Investigating this shift in equilibrium and the conserved and evolving networks that may govern it could provide insights into how conformational ensembles are regulated in response to post-translational modifications. Interestingly, conserved phosphorylation sites S347 in caspase-8, S150 in caspase-3, and T173 in caspase-7 are located in the same spatial position near α-helix 3 (Figure 1B) of the catalytic subunit and are highly conserved across organisms [18,19]. Phosphorylation at this hotspot has been shown to reduce activity for caspase-8, -7, and -3 [20,21]. Moreover, phosphorylation of S305 in caspase-8 (Figure 1B), although not conserved and located near the hotspot, appears to leverage this allosteric hotspot to modulate function [22]. Additionally, ubiquitination of caspase-8 at K224, K229, and K231 (Figure 1B) and highly conserved residues K351 and K353 in cellular FLICE-like inhibitory protein (cFLIP) regulates activation and degradation by altering interaction networks in loop regions at the base of the structure [23]. Similarly, ubiquitination of highly conserved residues K351 and K353 in cFLIP (Figure 1B) at the base of the structure suggests a parallel regulatory mechanism [24]. Lastly, zinc binding in loop regions at the base of caspase-6 (Figure 1B) allosterically inhibits function by inducing a coil-to-helix transition in the β-sheet above helix 3 [25]. Together, these findings strongly suggest that present-day caspases have repurposed inherited allosteric hotspots and regulatory networks at the base of the structure from a common ancestral scaffold to modulate function at distant active-site loops by altering conformational equilibrium.

In order to characterize conserved amino acid networks and their evolution in the conformational landscapes of caspase subfamilies, we employed ancestral protein reconstruction (APR), molecular dynamics (MD) simulations, essential dynamics [free energy landscape(FEL)], network analysis, and mutational *in vitro* folding studies [26–28]. Invertebrate sequences were removed from our APR because they are poorly described, and previous studies reveal that the folding patterns are under distinct evolutionary forces [9]. The presence of caspases-3, -6, -7, and -8-like homologs in non-chordates suggests that a pool of ancestral sequences acquired from the evolution of a common ancestor of Metazoa around 700 mya provided the framework for the evolution of their extant homologs in chordates [11,28]. To simulate the ancestral pool, we reconstructed a pool of ancestral caspases (Figure 1C) named ancestor of all (AOA-1, -2, -3) using chordate caspase sequences from three databases (Supplementary File S1). Furthermore, we reconstructed the ancestral sequences of all initiators (AOI-1, -2), ancestor of caspase-8/-10 (Anc8/10), and ancestor of cFLIP (Figure 1C) from an additional initiator caspase database (Supplementary File S1). Ancestor of all effectors (AOE-1, -2), ancestor of caspase-3/-7 (Anc3/7), and ancestor of caspase-6 (Anc6) were reconstructed from previous databases compiled by Grinshpon et al. [28] (Figure 1C). A sequence alignment of all the reconstructed ancestral sequences with the extant human caspase sequences used in this study is shown in Supplemental Figure S1.

We performed MD simulations in water and in urea with the active (intersubunit linker cleaved), dimeric (intersubunit linker intact), and monomeric (NMR solution structure of caspase-8: 2K7Z) structures (Figure 1D) acquired from the protein data bank (PDB) or modeled according to the nearest neighbor in the evolutionary tree (described in detail in the methods section). The NMR structure of caspase-8 (PDB ID: 2k7z) is the only available structure of a monomer; hence, we utilized it to model all of the sequences [29]. Given the conserved free energy of the monomer in both subfamilies and the congruence of previous MD simulations with experimental findings, the 2k7z caspase-8 monomeric conformation stands as robust and ideal for modeling all the sequences [15].

Our data show that both monomeric and dimeric conformations exhibit an expansive ensemble of structures in contrast with the active conformation. MD simulations in water and in urea reveal that the small subunit has subfamily-specific dynamics that predate the caspase common ancestor in chordates. These studies provide insight into the energetic barriers that trap initiators in monomeric conformations while guiding effectors toward dimeric conformations as a result of amino acid interactions in the small subunit. Moreover, with in-depth network analysis of MD simulations, we systematically categorized stable scaffolding interactions within the hydrophobic core, and we show that the interactions have remained highly conserved since the common ancestor in all apoptotic caspases. In addition, we identify a network of residues in the small subunit with varied biochemical properties that are specific to the subfamilies. *In vitro* folding analyses with the ancestral mutations swapped into subfamilies of extant caspases reveal the network to be critical to the evolution of the protein fold and dynamics, beyond the identified stable core in apoptotic caspases. Limited trypsin proteolysis followed by mass spectrometry shows that the mutations in the small subunit of the ancestral caspases affect the stability of an allosteric hotspot via a network of amino acid interactions in the loop regions at the base of the structure. Furthermore, we identify critical conserved residues in these networks that are located at the allosteric hotspot (Figure 1B) and that control conformational dynamics and fold beyond a conserved folding intermediate. The conserved amino acid network encompasses the stable conserved core in all apoptotic caspases. Lastly, we show that a network of conserved phenylalanine residues mediates allosteric signals to effect side chain packing in the hydrophobic core and hence affect global dynamics and caspase conformations.

## Results

### Conformational stability of apoptotic caspases in chordates is governed by varied packing of amino acid side chains between helices and β-sheets

We examined the stability of caspases using amino acid interaction networks [26]. In network analysis, amino acid residues are represented as nodes, and their interactions with neighboring residues are called edges [30]. Specifically, we utilized the degree centrality (DC) metric, which quantifies the number of non-covalent interactions that residues make with their neighbors [26]. The conformational dynamics of monomeric, dimeric, and active states are influenced by the arrangement and organization of residues in the core [31–33]. Utilizing degree metrics, we analyzed the spatial distribution of non-covalent bonds within distinct caspase conformations across the entire tree (Figure 1C and D), providing insights into their evolution spanning a significant temporal scale of 500 million years [34].

**Figure 2:**
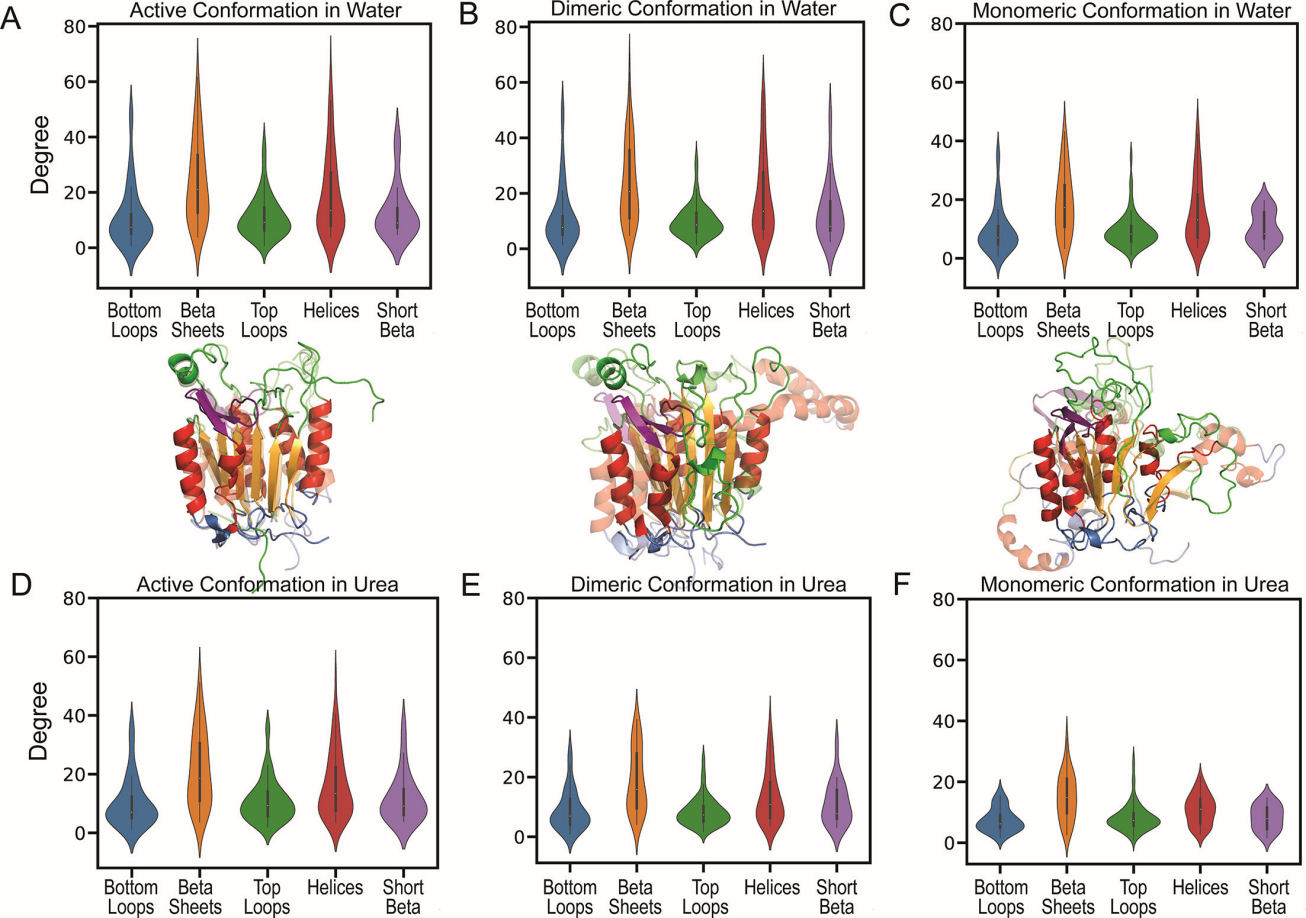
Average degree data in water and in 8M urea. Violin plots representing the average degrees/contacts for the (**A**) active, (**B**) dimeric, and (**C**) monomeric conformations in water and (**D**) active, (**E**) dimeric, and (**F**) monomeric conformations in 8M urea. Bottom loops (blue), β-sheets (orange), catalytic site loops (green), helices (red), and the short β-sheets (purple) are color-coded on the modeled caspase-8 structures. The solid structures represent the caspase-8 in water, and the translucent structures represent caspase-8 in 8M urea.

The average degree data for each conformation, obtained from MD simulations in water, were subdivided into helices,β-sheets, and loops, with loops further classified as top loops if located in the active site region and bottom loops if positioned at the base of the structure; an additional β-sheet structure observed in crystal structures of caspases above helix 3 was defined as a short β-sheet. Violin plots were used to display the distribution of degree values for the active (Figure 2A), dimeric (Figure 2B), and monomeric (Figure 2C) conformations, providing a detailed statistical view that captures both central tendencies and the spread of non-covalent interaction patterns across the secondary structure elements over evolutionary time. The violin plots for the monomeric conformation (Figure 2C) show a considerable difference for the β-sheets and α-helices compared with the dimeric conformation (Figure 2B), indicating that the transition from monomer to dimer requires extensive rearrangements in these regions. In contrast, comparison of dimeric and active conformations shows smaller alterations, suggesting modest rearrangements in the transition from dimeric to active. Furthermore, sub-classification of the degree scores into three categories based on ConSurf conservation scores (Supplementary Figure S2)—high conservation (score of 9–8), intermediate conservation (score of 7–6), and variable (score below 5)—shows that the conserved and intermediate conserved residues make the most contacts (non-covalent bonds) among the categories (Supplementary Figure S2 A,B,C) [35].

Similarly, we plotted degree data as violin plots for MD simulations in urea. The comparison of the violin plots for simulations in water (Figure 2A–C) to those in urea (Figure 2D–F) reveals that monomeric (Figure 2C and F) and dimeric (Figure 2B and E) conformations lose substantial non-covalent interactions, the majority of which are from loss of contacts in α-helices andβ-sheets. In addition, these plots show that the stability is mostly dictated by contacts in the β-sheets and helices that reside in the hydrophobic core. Moreover, sub-classification suggests that conserved and intermediate-conserved residues display the maximum change for simulations in urea Supplementary FigureS2, panels D,E, and F in comparison with those in water Supplementary FigureS2, panels A,B, and C.

Altogether, the data show that the stability of caspase conformations across a ~500-million-year evolutionary span is significantly influenced by non-covalent interactions between β-sheets and α-helices, which is not surprising. However, when these interactions are subdivided, interestingly, maximal non-covalent interactions in the dimeric and active conformations are formed by highly conserved residues. While one might assume from the superposition of cartoon representations that the protein core remains largely unchanged across monomeric, dimeric, and active conformations, our studies reveal extensive rearrangements in the core interaction networks. Notably, the monomeric conformation makes the fewest non-covalent bonds between helices and β-sheets, followed by a dramatic increase in the inactive dimeric conformation. Caspase dimerization is shown to contribute to a ~two-fold increase in the free energy of the monomer, and our results suggest that this increase is not simply a result of interface rearrangements in the small subunit to form the dimer but rather a function of global amino acid side chain rearrangements in the helices and β-sheets [14–16,36]. The conformational free energy of the monomeric and dimeric inactive states correlates well with contact loss, offering insights into the high stability of the active conformation. We note that the conformational stability of the active dimer is not known because folding is irreversible when the chain is cleaved.

### The small subunit displays conserved patterns of stability across initiator and effector subfamilies in monomeric and dimeric conformations

Since the active state is very stable and doesn’t display varied dynamics in our simulations, we characterized the evolution of the FEL of the dimeric and monomeric conformations of all the extant and ancestral caspases (Figure 1C) [37]. The essential dynamics were identified using principal component analysis (PCA) based on MD simulations in urea and in water [38]. The first two principal components (PCs), which explain much of the variance in the data, were utilized to generate an FEL for evolutionary analysis.

**Figure 3:**
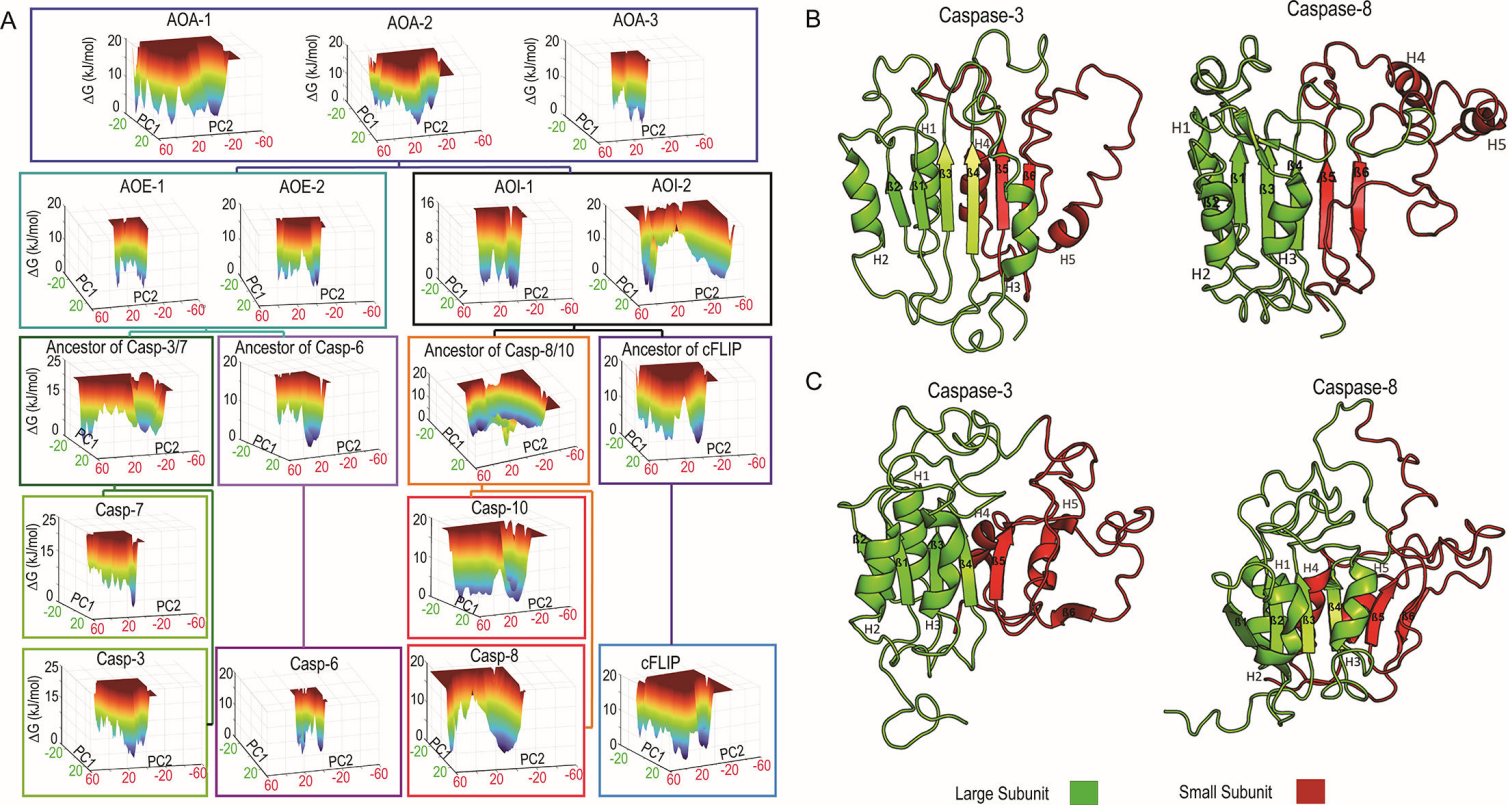
The free energy landscapes of the dimeric conformation of caspases in urea obtained from 200 ns MD simulations. (**A**) The free energy landscapes (FELs) are generated as a function of projections of the MD trajectory onto the first (PC1) and the second (PC2) eigenvectors, respectively. Target FEL minima are represented as the metastable states visited during the simulations in urea for the dimeric conformation of (**B**) caspase-3 and caspase-8. Target FEL minima in the monomeric conformation for simulations in water are represented as the metastable states in (**C**) caspase-3 and caspase-8.

Comparing the FEL in urea (Figure 3A) to that of water (SupplementaryFigure S3), a larger conformational space in urea is observed, indicating enhanced sampling of atoms. Despite simulation times of 200 ns in 8M urea being insufficient for complete unfolding, these investigations can demonstrate relative stability across caspases, with a wider landscape indicating more unfolding. Moreover, the area or the width occupied by the FELs correlates with experimental findings. In the effector subfamily, the folding landscape of the dimeric conformation has been extensively studied [14,16,36]. Among the members of this subfamily, caspase-6 has been found to be the most stable when compared with caspase-3, caspase-7, and AOE-2. This observation is consistent with our findings, which indicate that caspase-6 exhibits the least accessible conformational space, as shown in Figure 3A, in comparison with caspase-3, caspase-7, and AOE-2. Currently, there are no folding data to quantify the conformational free energy of the dimeric conformation of caspases in the rest of the evolutionary tree, which limits our ability to provide a comprehensive evolutionary perspective. However, since our MD simulations in urea in the current and previous studies show excellent agreement with experimental findings, the data provide us an avenue to characterize the dimeric conformation in regard to an overall evolutionary perspective. Data from FEL plots (Figure 3A) suggest that the effector caspase lineage utilized the AOA-3 scaffold, with narrow FEL, from the ancestral pool, leading to a narrow FEL from AOE-1, -2 to the extant caspases-3, -6, and -7, in essence stabilizing a dimeric scaffold in the conformational ensemble. In contrast, the initiator lineage evolved to exhibit a wider FEL from AOI-1 and -2 to the extant caspases-8, -10, and cFLIP. Note that in the effector caspase lineage, only the Anc3/7 shows a wide landscape, which is discussed later in this section.

Given the insights garnered from FEL of simulations in urea, which indicate distinct stability profiles among caspase subfamilies in their dimeric conformation, we further analyzed the last metastable state that these systems visited to determine whether we could distinguish similar patterns of destabilization in each caspase subfamily [39]. In Figure 3 (panels B and C), we show examples of metastable states of caspase-3 and caspase-8 extracted from the FEL (Figure 3A), which correspond to the last observed metastable state in urea for effector caspase-3 and initiator caspase-8, in dimeric conformation. The small subunit of caspase-3 (Figure 3B) unfolds to a lesser extent than the small subunit of caspase-8 (Figure 3C), and this trend is observed on average in the effector and initiator tree, respectively (Supplementary Figure S4). Hence, the broader landscapes observed in Figure 3A for initiator caspases correspond to a less stable small subunit in the dimeric conformation. In caspase-3 (Figure 3B and Supplementary Figure S4), cFLIP, and other ancestors (Supplementary Figure S4), helices 2 and 3 detach from the β-sheets. Note that the anti-parallel β-sheet 6 is destabilized in the Anc3/7 (Supplementary Figure S4), resulting in the destabilization of the small subunit, which gives rise to the larger FEL shown in Figure 3A and is an exception in the effector caspase lineage. In the context of evolution and natural selection, it is conceivable that caspase-3/7-like sequences may have emerged within the ancestral gene pool through gene duplication without adversely affecting the fitness of the ancestral organism. This could have subsequently paved the way for neofunctionalization in caspase-3 and caspase-7. These results highlight the trial-and-error nature inherent in evolution and natural selection, shaping the design of specialized enzymes across extant organisms.

For the monomeric conformation in urea, there is no discernible difference in the FEL between initiators and effectors (Supplementary Figure S5). However, in these simulations with the same urea concentration (8M), the FEL for the monomeric conformation (Supplementary Figure S5) is broader (PC1) than the dimeric conformation (Figure 3A), indicating extensive unfolding of the less stable monomeric conformation. Interestingly, for simulations in water, the average MD conformation reveals that β-sheet 6 unfolds in caspase-3, but the monomeric conformation of caspase-8 is relatively stable (Figure 3C), which is observed to be an average trend in the effector and initiator subfamilies (Supplementary Figure S6), respectively.

In summary, conformational landscapes of monomeric and dimeric caspases were already established in the common chordate ancestor, and their variable stability has been passed on faithfully in each subfamily. Simulations of the entire caspase tree in urea suggest that the dimeric conformation of the initiator caspase lineage is less stable compared with the dimeric conformation of the effector caspase lineage. The data suggest that the lower stability is due to weakened interactions between helices 4 and 5, and β-sheets 5 and 6. Conversely, simulations of caspases in water reveal that the effector caspase lineage is unstable in the monomeric conformation, particularly due to destabilizing interactions near β-sheet 6. Hence, thermodynamic insights suggest that effectors exist as dimers since the monomeric conformation in their ensemble is unstable, while in initiators, the monomer is more stable and may present an energy barrier to dimerization; moreover, even after initiators dimerize on platforms, the resulting dimeric state remains less stable than in effectors. Overall, these differences can be attributed to the differential evolution of amino acid interactions in the small subunit, which are largely responsible for changes in the conformational landscape of the initiator and effector subfamilies.

### In caspase subfamilies, the small subunit and co-evolving networks of residues evolve folding landscapes while also demonstrating an implicit connection to an allosteric hotspot

**Figure 4:**
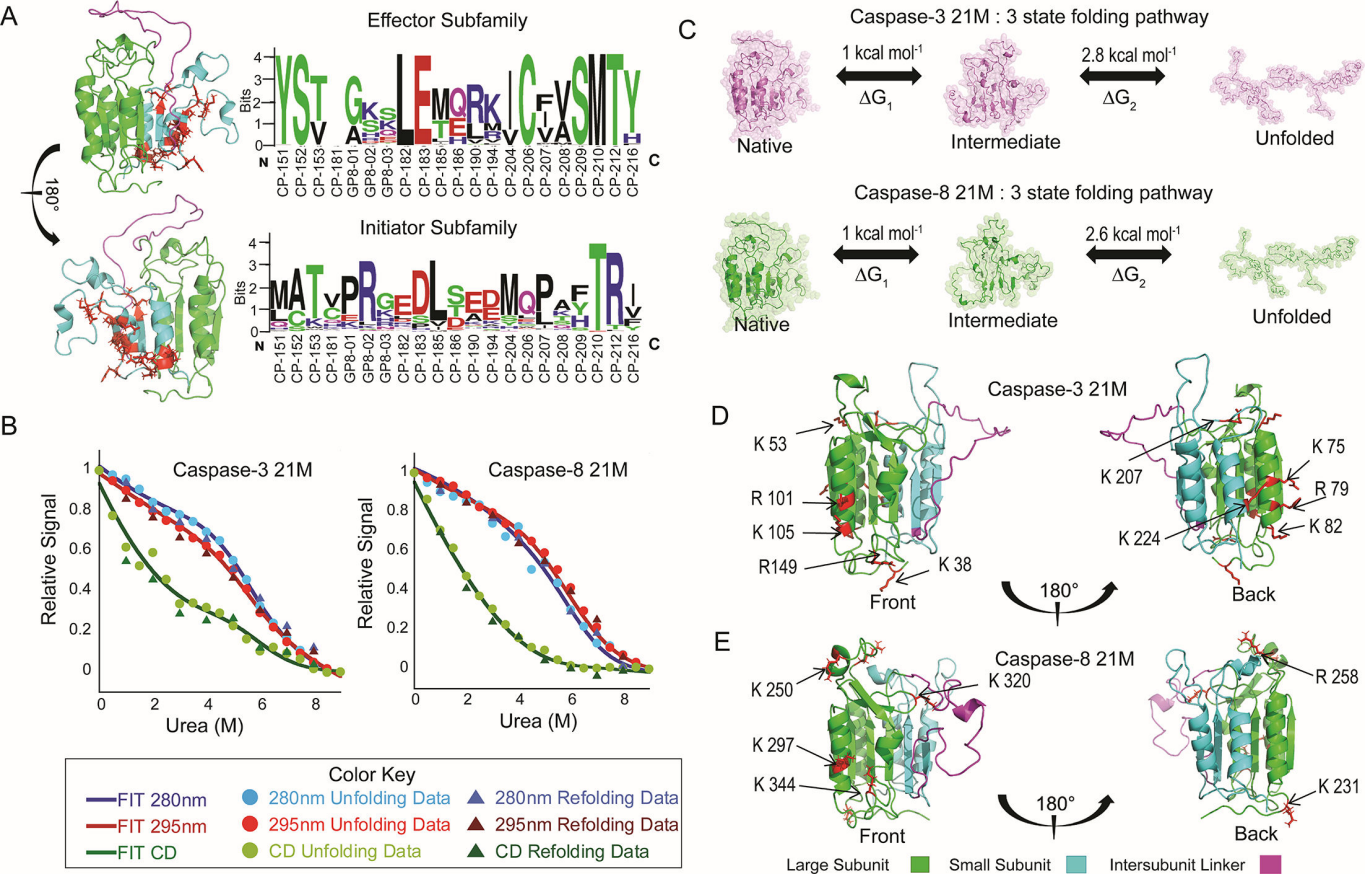
Stability of caspase network mutants. (**A**) Site-specific amino acid differences within subfamilies in the small subunit represented on a web logo and mapped onto the caspase-8 monomer. The large subunit is depicted in green, the small subunit in cyan, the inter-subunit linker in magenta, and site-specific differences are mapped as red sticks onto the structure. (**B**) Equilibrium unfolding of caspase-3 network mutant and caspase-8 network mutant at pH 7.5 monitored by fluorescence emission with excitation at 280 nm (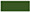), 295 nm ( 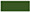), CD (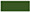), and refolding at 280 nm (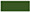 ), 295 nm (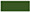), and CD (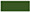). As described in the text, solid lines represent global fits to the data at 280 nm (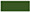), 295 nm (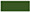), and CD (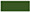). (**C**) Representation of the free energies obtained from the global fits for each step of unfolding for caspase-3 network mutant and caspase-8 network mutant. Cleavage products of limited trypsin proteolysis at pH 7.5 were determined by MALDI-TOF MS as described in the text and represented on caspase-3 network mutant structure (**D**) and caspase-8 network mutant structure (**E**). The large subunit is depicted in green, the small subunit in cyan, the inter-subunit linker in magenta, and the cleavage sites as red sticks.

We identified residues within the small subunit that display distinct biochemical properties between the initiator and effector subfamilies in the entire chordate database (SupplementaryFile S1). These 21 residues (Figure 4A) map to the small subunit, as depicted by the caspase-8 structure in Figure 4A and the alignment in Supplementary Figure S1. We hypothesized that these 21 residues influence the observed conformational diversity among caspase subfamilies. We reasoned that by exchanging the 21 mutations (21M) in caspase-3 and caspase-8, it might be possible to influence the monomer–dimer equilibrium in caspases-8 and -3.

We undertook *in vitro* folding investigations with these 21M in caspase-8 and caspase-3 to test the veracity of our idea. We tracked urea-induced unfolding and refolding of tertiary structure using excitation wavelengths of 280 nm (all aromatic residues) or 295 nm (tryptophan residues only) and fluorescence emission from 300 nm to 400 nm. We also performed far-UV circular dichroism for 21M caspase-3 (Supplementary Figure S7 A,B,C) and 21M caspase-8 (Supplementary Figure S7 D,E,F) to monitor changes in secondary structure. Our folding studies revealed no dependence on the protein concentration for caspase-3 21M or for caspase-8 21M(Figure 4B). The findings suggest that the equilibrium of monomer-to-dimer in caspase-3 was shifted to the monomeric state. However, the comparable mutations did not result in a stable dimer for caspase-8. Unexpectedly, the folding data for caspase-3 21M and caspase-8 21M show that the folding patterns and thermodynamic parameters exhibit a remarkable similarity (Figure 4B), and datasets for both mutants can be characterized by a three-state folding model for a monomer (Figure 4C), in which the native monomer unfolds to a partially folded intermediate prior to unfolding (N ↔ I ↔ U). For both proteins, the mutations result in substantial changes in stability compared with their wildtype (WT) counterparts [15,16]. The thermodynamic parameters extracted from the fits of the data (Supplementary Table S1) show that the first transition (N ↔ I) exhibits a conformational free energy of ~1 kcal/mol, while the second transition (I ↔ U) exhibits a conformational free energy of ~2.7 kcal/mol. Thus, both caspases with 21M in the small subunit exhibit a total conformational free energy of ~3.7 kcal/mol.

We reasoned that the mutations destabilize the small subunit due to interrupting the networks of co-evolving residues in the large subunit. Consequently, these 21M proteins became confined to an evolutionarily conserved, partially stable, folding intermediate. Overall, the mutations appear to render the proteins incapable of accessing other conformations within their respective ensembles, as opposed to their WT counterparts which fold into a stable dimer (caspase-3) or monomer (caspase-8). To further identify regions that were destabilized because of the 21M mutations, we performed limited trypsin proteolysis and used MALDI-TOF mass spectrometry to identify regions of the proteins with the highest cleavages at a time point of 1 h. The results were compared for WT caspase-3 (Supplementary Figure S8 A), caspase-3 21M (Supplementary Figure S8 B), and caspase-8 21M (Supplementary Figure S8 C). Notably, identical conditions to those described in our previous report were used for caspase-8 WT; consequently, we compared the results of the 21M mutants to WT from our previous investigations for caspase-8 [15]. We compared the top ten cleavages for caspase-3 WT (Supplementary Figure S8 D) and caspase-3 21M (Supplementary Figure S8 E) and for caspase-8 WT (Supplementary Figure S8 F) and caspase-8 21M (Supplementary Figure S8 G) to determine regions that are destabilized in their 21M versions. Overall, the data for caspases-3 and -8 (Figure 4D and E, respectively) indicate that helices 2 and 3 and loop regions in the large subunit that are otherwise inaccessible to the protease in the WT proteins are destabilized in the 21M variants. These findings indicate that mutants in the small subunit affect the stability of helices 2 and 3 in the large subunit, with a particular clustering near an allosteric hotspot. The results suggest that interactions in co-evolving networks of amino acids link the small subunit to the allosteric site.

### Highly conserved residues that exhibit high degree centrality and betweenness centrality provide a scaffold for the evolution of conformational dynamics in the subfamilies

In order to identify co-evolving networks and explore the inherent link between the small subunit and the allosteric pocket on the large subunit, we first classified conserved networks in the central core, as well as other evolving networks in the protein sequences. We employed DC and betweenness centrality (BC) metrics to discern critical elements in the networks [26]. While DC reflects how extensively a residue is connected within the network, BC quantifies the extent to which a residue serves as a bridge along the shortest paths linking distant regions of the protein, highlighting its potential role in mediating allosteric communication [40]. Nodes with higher BC values tend to occupy topologically strategic positions, acting as bottlenecks or relay points that facilitate efficient signal propagation across structurally or functionally distinct regions [41]. These metrics together allow us to identify residues with numerous interactions and to distinguish interactions that may be central to communication pathways within the protein structure.

**Figure 5:**
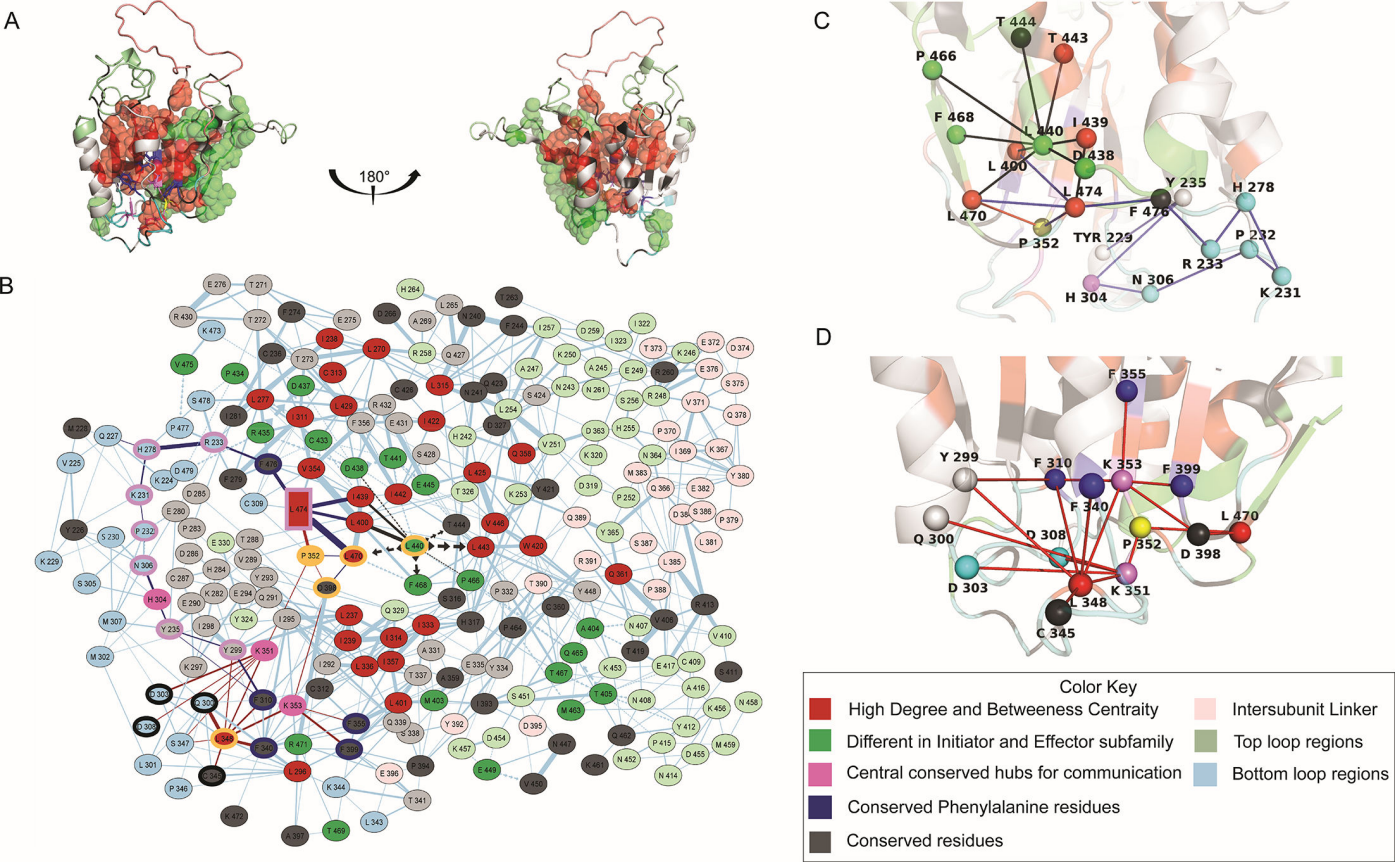
Degree and betweenness centralities in caspases. (**A**) MD average structure of monomeric caspase-8 in water highlighting residues having a high degree and betweenness centrality as red spheres, residues that are different within the initiator and effector subfamilies as green spheres, and the conserved phenylalanine residues in the core as blue sticks. Residues are numbered according to the CaspBase scheme (50) for cross-species and ancestral comparison. (**B**) A 2D representation of the network map of monomeric caspase-8 in water highlighting conserved residues as gray nodes, residues on the inter-subunit linker as light pink nodes, residues on the top loops as light green nodes, and residues in the bottom loops as light blue nodes. Interaction networks mapped onto the caspase-8 monomeric structure for residues on the bottom loops at the back (below helices 1 and 4) (**C**) and residues on the bottom loops on the front (below helices 2 and 3) (**D**).

To identify nodes in identical spatial positions that display high values for these metrics across evolutionary time, we averaged positional values across the entire tree for both the monomeric and the dimeric conformations separately. The active conformation was excluded from the analysis due to its inherent stability and limited dynamical behavior. Pair plots of degree vs. BC for the average in the dimeric (Supplementary Figure S9 A) and monomeric (Supplementary Figure S9 B) conformations show that most values cluster below 20 for degree and 1000 for betweenness. Residues that fall outside this quadrant in both the monomeric and dimeric conformations represent stable scaffolding interactions around which conformations fluctuate [42]. We mapped high DC and BC residues in all caspases onto average weighted network maps obtained from MD simulations for both monomeric and dimeric conformations of caspase-3 (Supplementary Figure S9C and S9D) and of caspase-8 (Supplementary Figure S9E and S9F). The data demonstrate pronounced thick edges, extensive interconnections, and central roles for each high DC and BC amino acid within the entire network of amino acid interactions. Subsequently, the residues were mapped onto the structure of caspase-8 (Figure 5A), and predominantly occupy positions within the hydrophobic core. The results highlight the integral role for these residues in facilitating information transfer throughout the entire molecular structure. These residues belong to the intermediate and well-conserved groups (Supplementary Figure S1A and S1B) in the ConSurf grouping described above. The intermediate well-conserved residues predominantly populate the region of the molecule near helices 2 and 3. The less selective evolutionary pressure on these residues likely results in variable stability of helices 2 and 3 observed in some caspases in our MD simulations (Supplementary Figure S4). On the contrary, high DC and BC residues on the opposite side of the structure are highly conserved. Interestingly, in MD simulations of unfolding in urea (Supplementary Videos 1; 2), observed in reverse, the highly conserved residues are the first to collapse or fold, followed by the intermediate conserved residues. As conformations vary beyond the monomer, the highly conserved residues exhibit minimal dynamics, followed by the intermediate well-conserved, and then the other elements around them are prone to maximal dynamics.

The 21M mutations in the small subunit (Figure 4A) are absent from the high DC and BC set of residues (Figure 5A and Supplementary Figure S1), indicating that they are dynamic as conformations vary. In the network of the 21M in caspase-8, L440 (at the bottom of helices 4 and 5) acts as a central hub (Figure 5, panels B and C), making the most stabilizing contacts at the bottom loops, and is highly conserved in all initiator caspases (Figure 4A and Supplementary Figure S1). Moreover, research findings indicate that interactions within the bottom loop region contribute significantly to dimer stability, accounting for 30–50% of dimer stability [43]. The shortest pathway for mutations around the L440 hub to affect the allosteric hotspot is facilitated by a critical residue, P352, located on β-sheet 4, which is also, on the other end, in the active site loops is the location of the catalytic cysteine (Figure 5B and C). Proline can adopt cis and trans isomers, and the highly conserved P352 is at a critical location that links high DC and BC networks located on one side of the protein to those on the other side. Furthermore, P352 is covalently connected to highly conserved K351 and K353, both of which bury into the hydrophobic core at the front face of the protein (Figure 5B and D). Hence, changes to the interaction network of P352 affect the packing of residues on the opposite face of the protein. This phenomenon is exemplified in morph videos featuring the monomeric and dimeric conformations of caspase-8 (Supplementary Videos 3; 4). Notably, as conserved tyrosine Y226 (N-terminus) packs into the bottom loop region in the rear, P352 is altered, impacting the arrangement of high DC and BC residues at the front (Supplementary Videos 3; 4). Thus, the data show that an alternate route to destabilize helices 2 and 3, and subsequently the allosteric pocket, involves a distinct path that includes changes to the positioning of conserved K351, K353, and P352 (Figure 5B and 5D).

In summary, by employing degree and BC measures, we identified a conserved network of non-covalent interactions between β-sheets and α-helices (Figure 1 and Supplementary Figure 1), which are central to stability and communication. Given that the folding and stability patterns of caspase-3 21M and caspase-8 21M converge (Figure 4B and C), this convergence may be a result of conserved elements spanning over 500 million years of evolution, corresponding to the identified high DC and BC residues (Figure 5A). Our network analysis suggests that the 21M in the small subunit ultimately destabilize co-evolving networks at the base of the structure that, in turn, affect the allosteric pocket through destabilizing helices 2 and 3 on the opposite face of the protein. Furthermore, subtle modification of core residues around helices 2 and 3 allows for varied allosteric regulatory mechanisms in apoptotic caspases. This evolving allosteric network suggests differential modulation of catalysis in apoptotic caspases by affecting the critical histidine above helix 3 and dimerization via the small subunit in ways unique to each caspase.

### Conserved residue hubs at the base of the structure control the active-site cysteine and histidine dyad while also regulating conformational dynamics

Conformational alterations driven by global shifts in residue interaction networks are frequently modulated by critical hub residues that govern conformational landscapes and allosteric signaling in proteins [44–46]. Here, we observed that all of the communication signals from the small subunit that alter helices 2 and 3 traverse bottom loop regions and appear to mainly modulate conserved residues H304, K351, and K353. The modulations create global rearrangements in the protein structure and may be involved in trapping the conformational ensemble of the 21M mutants in the partially folded state described above. To examine these residues further, we performed alanine scanning mutations of these residues in caspase-8 and characterized changes in the folding landscape.

**Figure 6:**
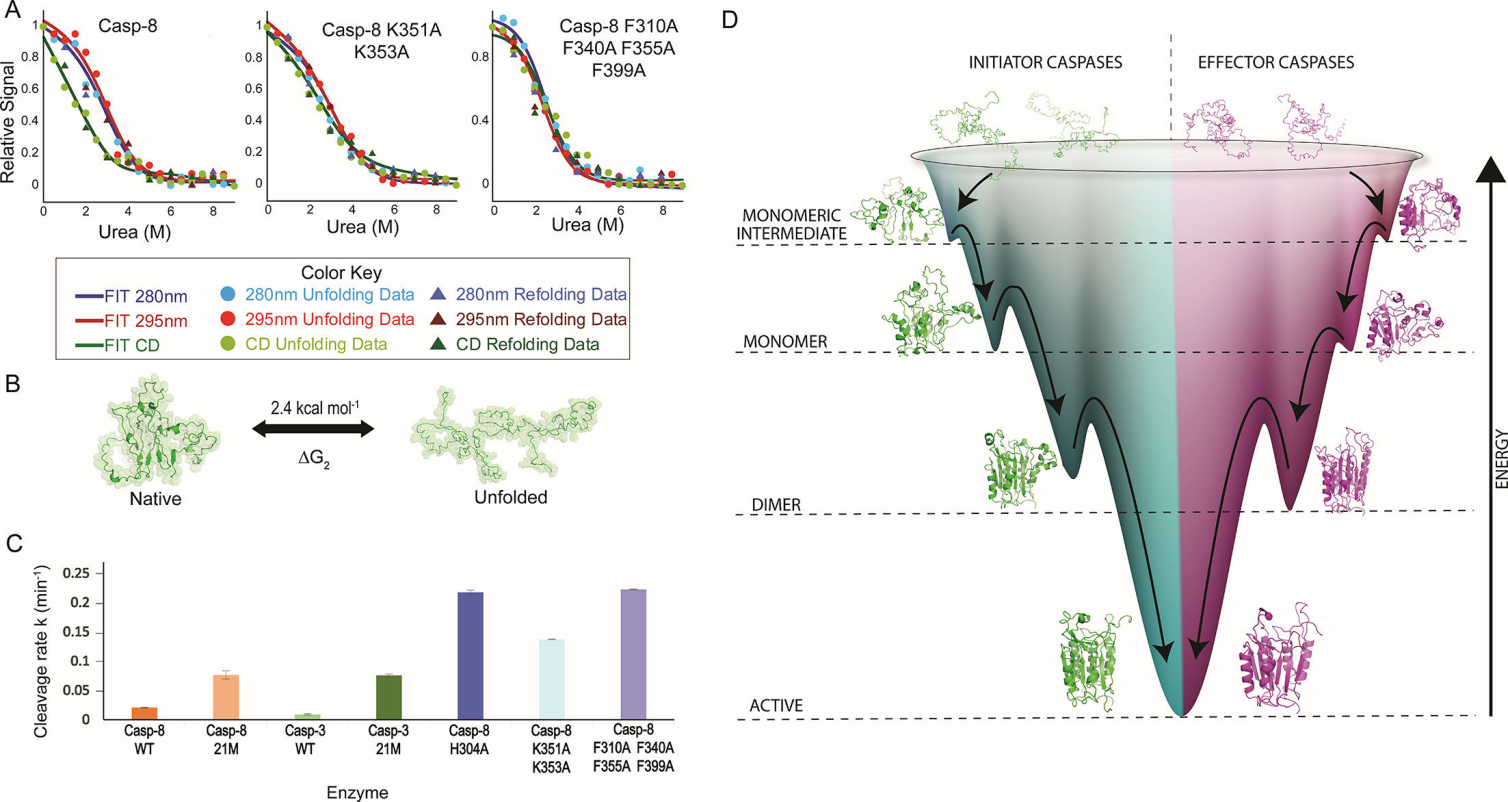
Caspase free energy landscape. (**A**) Equilibrium unfolding of caspase-8 H304A, caspase-8 K351A + K353A, and caspase-8 F310A + F340A + F355A + F399A at pH 7.5 monitored by fluorescence emission with excitation at 280 nm (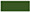), 295 nm (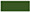), CD (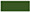), and refolding at 280 nm (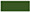), 295 nm (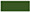) and CD (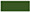). As described in the text, solid lines represent global fits to the data at 280 nm (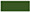), 295 nm (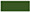), and CD (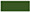 ). (**B**) Representation of a two-state unfolding model and free energy obtained from the global fits of unfolding for caspase-8 mutants. (**C**) Kinetics of cleavage of annotated bands from limited trypsin proteolysis (Supplemental Figures S8 and S11) were fit to single exponential equation to determine the apparent rate constant, as described in the text. (**D**) A folding funnel model for the conformational stability of caspases.

Similar to the 21M mutants (Figure 4B), described above, we examined urea-induced unfolding and refolding using fluorescence emission and circular dichroism spectroscopies for caspase-8 H304A (Supplementary Figure S10 A,B and C) and caspase-8 K351A + K353A (Supplementary Figure S10 D,E and F). The results show that the folding model shifts to a two-state pathway for caspase-8 H304A and K351A + K353A mutants (Figure 6A and B), suggesting that the native state observed in caspases-3 and -8 21M (Figure 4C) is destabilized in favor of the partially folded intermediate. The folding trends (Figure 6A) and the parameters (Supplementary Table S2) show that the conformational free energy of the intermediate seen in the 21M mutant (Figure 4C) is not affected. Altogether, the data suggest that replacing K351, K353, and H304 with alanine destabilizes caspase-8 such that the ‘native’ conformation is similar to the partially folded intermediate (I) observed in the 21M variants.

After classifying high DC and BC residues, we identified highly conserved F310, F340, F355, and F399 that, despite being in the hydrophobic core near the allosteric pocket (Figure 5D), do not appear among the high DC and BC residues, indicating their dynamic behavior as conformations vary from monomer to dimer. As the protein structure progresses from monomer to dimer to active conformations, these highly conserved phenylalanine residues appear to transmit changes at the bottom loop regions to the active site by rearranging the high DC and BC side chains in the core (Supplementary Videos 5; 6). Hence, we hypothesized that mutating these residues would not affect the stability of the partially folded intermediate observed in the 21M mutants (Figure 4C), or as we speculate, the comparable ‘native’ state caspase-8 H304A and K351A + K353A variants (Figure 6A). We mutated the four phenylalanine residues to alanine and determined changes in the folding and stability of the protein (Supplementary Figure S10 G, H, and I). Surprisingly, caspase-8 (F310A, F355A, F355A, F399A), called caspase-8 (F/A), exhibited an increase in secondary structure compared with the H304A or K351A/K353A variants (Supplementary TFigure S10 C,E and F). For the protein with the F-to-A mutations, the data are well described by a two-state folding model (N ↔ U) with a conformational free energy of 2.3 kcal/mol (Supplementary Table S1), which is identical with those of the H304A and K351A/K353A caspase-8 mutants. Together, the results agree with our hypothesis, suggesting that the four phenylalanine residues have similar effects on the native structure as H304, K351, and K353 by destabilizing the native conformation relative to the partially folded intermediate. However, since the residues have fewer contacts, as determined by lower DC and BC values, their role likely involves varying conformational stability through modulating the packing of side chains in the hydrophobic core.

We examined the caspase-8 variants by limited trypsin proteolysis coupled with mass spectrometry, and we show that the allosteric pocket is destabilized in caspase-8 H304A (Supplementary Figure S11 A and B), K351A + K353A (Supplementary Figure S11 E and F), and F310A + F340A + F355A + F399A (Supplementary Figure S11 E and F). Furthermore, the rate of cleavage of the native protein band correlates with our folding models. For example, caspase-8 21M and caspase-3 21M, both of which unfold via a three-state pathway, are cleaved at a similar rate, albeit at a higher rate than their WT counterparts. The data also show that caspase-8 H304A, caspase-8 (K351A + K353A), and caspase-8 (F/A), which unfold via a two-state pathway, are cleaved at a higher rate than the 21M variants (Figure 6C). Overall, the data correlate well with our conclusion that mutations affecting the packing of the hydrophobic core or the conserved interaction network destabilize the native conformation of the monomer such that the protein is partially folded.

Overall, our studies suggest that the high DC and BC residues collapse early in refolding and substantially contribute to the stability of the partially folded intermediate in the folding transition of U to I (Figure 4C). In addition, the high DC and BC residues have been conserved for more than 500 million years, with little modification in the intermediate conserved region near the allosteric pocket (Supplementary Figure S1 A and B ). In caspase-8, H304, K351, and K353 are part of a conserved interaction hub, and alterations around the hub are sensed by several phenylalanine residues (F310, F340, F355, and F399), which modulate packing in the hydrophobic core. We analyzed the crystal structures for the ancestor of effector caspases [28] and the Anc6 [28] with eMap [47], and the results suggest that the highly conserved phenylalanine and cysteine residues in the core serve as electron or hole hopping channels in caspases to transport charges rapidly over long distances (Supplementary Figure S12) [47,48]. We suggest that caspases might use these transfer channels to modulate enzyme activity through changes in conformational dynamics. Lastly, K351, P352, K353, and F355 are located on β-sheet 4, which carries the catalytic cysteine, thereby making this strand a vital element in catalysis and conformational regulation. The highly conserved H304, which modulates helices 2 and 3 in the allosteric pocket, plays a crucial role in regulating the position of the catalytic histidine. Collectively, our study provides mechanistic insights into allosteric control mechanisms that influence catalysis and regulate the conformational diversity within apoptotic caspases.

### Discussion

The existence of multiple apoptotic caspases such as caspase-3, -6, -7, -8, -10, and pseudo-caspases like cFLIP, which acquired the same fold but lost catalytic function, reflects an evolutionary strategy to diversify regulation and function within the apoptotic machinery [11]. Although structural data reveal a shared ancestral fold, they are limited to static, highly superimposable snapshots and fail to capture how these proteins move and function dynamically over time. These motions are often complex, involving multiple variations in structural states, but thermodynamic concepts such as the energy landscape offer a rational framework to describe and interpret this conformational diversity. MD simulations can provide insights into local motions near low-energy basins, but they often fail to capture high-energy intermediate states. Although enhanced sampling strategies can reveal such intermediates, they are rarely paired with experimental insights [49]. In this study, we integrated evolutionary tools with experimental and computational approaches to provide a dynamic picture of present-day apoptotic caspases, including high-energy intermediates, by in-depth exploration of how their conformational and folding landscapes evolved over time through both conserved and divergent features.

The folding funnel depicted in Figure 6D highlights our findings in conjunction with comprehensive experimental folding research conducted previously for initiator and effector caspase subfamilies [14–16,36]. Network analysis for simulations in water and in urea demonstrates that the active conformation is the most stable, followed by the dimeric and monomeric conformations, which is illustrated as energy gradients for these conformations in the folding funnel (Figure 6D). The active conformation depicts the lowest minima in the folding funnel (Figure 6D) and indicates maximum stability among all conformations. There are no experimental conformational free energy data for initiator caspases in the dimeric conformation; however, insights gleaned from our FEL analysis show that the dimeric conformation is less stable in initiator compared with effector caspases, resulting in a comparably lower minimum for initiator caspases in the folding funnel (Figure 6D). Simulations in water with the monomeric conformation suggest that effector caspases are less stable than initiator caspases due to a spontaneous loss of non-covalent interactions between the anti-parallel β-sheet and the core of the molecule, which has been represented by a higher energy barrier for the initiator caspases in the folding funnel model (Figure 6D).

We identified 21 residues in the small subunit that have evolved distinct biochemical properties within each subfamily. Swapping this network of residues between caspase-3 and caspase-8 did not switch their oligomeric states and folding pattern but caused both proteins to adopt a monomeric three-state folding pathway with a similar native-state free energy of 3.7 kcal/mol [14,15]. Moreover, the intermediate state in both mutants had a free energy of 2.7 kcal/mol, identical with that of the WT caspase-8 intermediate, suggesting the presence of a conserved intermediate that remains unaffected by these mutations [15]. This points to the evolutionary persistence of a shared folding intermediate across subfamilies. To understand what stabilizes this state, we combined network analysis of simulations in urea and water with sequence conservation, revealing residues that are central to communication, form persistent non-covalent interactions, and have remained largely unchanged over 500 million years, strongly suggesting their role in stabilizing the partially folded intermediate. Together, these findings position the ~2.7 kcal/mol intermediate as a conserved conformation within the conformational and folding landscape of all apoptotic caspases (Figure 6D).

Furthermore, mutations in conserved residues outside the resilient core, H304A and the K351A K353A double mutant (caspase-8 numbering) near the allosteric hotspot around helices 2 and 3, completely destabilize the native state, leaving a two-state pathway with equilibrium between the ubiquitous intermediate and the unfolded state. This suggests that these residues (H304, K351, K353) govern conformational equilibrium and influence dynamics beyond the stable core, which is likely the basis for regulation by post-translational modifications. Intriguingly, our studies identify a network of phenylalanine residues that appear to form a barrier between the bottom loops that host allosteric hotspots and the ubiquitous core that stabilizes the intermediate. The quadruple mutant F310A F340A F355A F399A (caspase-8 numbering) also shifts folding to a two-state pathway with the intermediate as the predominant conformation. It is interesting to note that conserved mutations in the bottom loops are more destabilizing to the native state and folding beyond the intermediate state than the 21M in the small subunit. This highlights the critical role of the bottom loop region in regulating the conformational ensemble beyond the conserved intermediate. Additionally, electron tunnel pathway analysis suggests that highly conserved residues in the bottom loops, along with core cysteines, may serve as an ancient relay system that enables rapid charge transfer from the allosteric pocket to the core or active site, efficiently transmitting allosteric modifications across the protein to affect global dynamics and active-site catalysis [48]. These insights not only make caspases an intriguing paradigm to further investigate electron tunnels in proteins but also provide a new avenue for investigating the mechanisms of allosteric communication in protein families and designing the next generation of small compounds for precise control of caspases.

## Materials and methods

### Ancestral protein reconstruction

To resurrect a highly probable sequence of the last common ancestral caspase of the chordates involved in the extrinsic pathway of apoptosis, we utilized a database of curated caspase sequences from CaspBase [50] that provided sequences from the initiator (caspase-8/-10/cFLIP) and the effector (caspase-3/-6/-7) subfamilies in the chordate lineage. A total of 600 sequences were obtained to generate three databases comprising 200 sequences each for APR. Representative taxa from various classes of Chordata (mammals, birds, fish, amphibians, and reptiles) were chosen in each database to resurrect three probable ancestral sequences (AOA1, AOA2, and AOA3). Since the pro-domain is subject to high sequence variation due to recombinations, insertions, and deletions, we pruned the sequences on Jalview [51] to remove the pro-domains after PROMALS3D structure-based alignment in PROMALS3D [52]. APR was carried out as previously described [28].

### Homology modeling

For MD simulations of caspases in the enzymatically active configuration, we used mature caspases from the PDB: caspase-3 (PDB ID 3DEI), caspase-7 (PDB ID 1K86), caspase-6 (PDB ID 3NKF), caspase-8 (PDB ID 3KJQ), AOE-1 (PDB ID 6PDQ), and pseudoenzyme cFLIP (PDB ID 3H11) [53–57]. The rest of the ancestral enzymes were modeled based on the nearest available mature caspase structure in the evolutionary tree for AOA-1,2,3 and AOI-1. The Anc3/7 and caspase-6 was modeled based on the crystal structures of caspase-3 (PDB ID 3dei) and of caspase-6 (PDB ID 3NKF), respectively. The Anc8/10 and caspase-10 were modeled based on the crystal structure of caspase-8 (PDB ID 3KJQ), and the ancestor of cFLIP was modeled based on the crystal structure of cFLIP (PDB ID 3H11). Procaspase enzymes from the PDB, exhibiting inactive but dimeric structures, were utilized for the dimeric conformation: procaspase-3 (PDB ID 4JQY), procaspase-7 (PDB ID 1K88), procaspase-6 (4N5D), and procaspase-8 (PDB ID 6PX9) [54,57–60]. Anc3/7, AOE-1,2, and of AOA-1,2,3 were modeled using procaspase-3 (PDB ID 4JQY) as a template, while Anc6 was modeled using procaspase-6 (PDB ID 4N5D) as a template. We used the procaspase-8 (PDB ID 6PX9) as a template for the entire initiator tree from AOI-1,2 to the extant caspases. For the monomeric configuration, we modeled the complete tree using the procaspase-8 structure (PDB ID 2K7Z), the only NMR solution structure available [29].

### Network analysis of amino acid interactions

To analyze the ancestral caspase networks, we utilized the open-source software Cytoscape [61]. Through SenseNet [26], a Cytoscape plugin, we visualized and allocated measures of importance to amino acids by converting MD interaction timelines into protein structure networks. The networks were analyzed for degree and BC for MD simulations performed in water and in 8M urea. Simulations were imported onto SenseNet, and input parameters were modified to examine Van der Waals, hydrophobic, and electrostatic interactions with a distance cut-off of 4 Å. The interaction weights were set to sum and the average in order to generate network interactions displaying the average DC and BC values for each residue derived from the entire simulation. Nodes displaying high DC and BC were further classified according to the conservation scores obtained from ConSurf [35]. All data files and additional illustrations related to degree and BC analyses are available on the associated GitHub page: https://github.com/Mithun-Nag/Evolution_of_Caspase_ensemble.git.

### eMap electron pathway analysis

All protein structures obtained from the PDB and used in this study, including caspase-3 (PDB ID: 3DEI), caspase-7 (PDB ID: 1K86), caspase-6 (PDB ID: 3NKF), caspase-8 (PDB ID: 3KJQ), AOE-1 (PDB ID: 6PDQ), pseudoenzyme cFLIP (PDB ID: 3H11), procaspase-6 (PDB ID: 4N5D), procaspase-8 (PDB ID: 6PX9), and the NMR solution structure of procaspase-8 (PDB ID: 2K7Z), were subjected to eMap analysis (https://emap.bu.edu/multiple) to identify shared electron and hole hopping pathways [47]. The proteins were used as inputs in the protein graph mining algorithm on the eMap web server to identify shared electron/hole hopping pathways among all the structures used in the study. Aromatic residues were selected for their electron transfer properties, along with cysteine and methionine, due to their roles as electron relay centers [62]. Conserved pathways were identified and mapped onto the structures of caspase-8 (PDB ID: 6PX9 and PDB ID: 2K7Z), revealing common electron transfer networks.

### MD simulations and free energy landscape

MD simulations for 200 ns were performed in water and in 8M urea for all caspases shown in Supplementary Figure S1. Each caspase was examined in three conformations: the active, the dimeric, and the monomeric conformations, as previously described [15]. To study the concerted motions of caspases and to identify the most significant motions in the simulations, PCA was conducted for all protein atoms in the trajectory. The PCs obtained from MD simulations in water and in urea are essentially the eigenvector values from the covariance matrix, each corresponding to a change in the trajectory. The eigenvalues and eigenvectors were analyzed using the gmx anaeig tool, and the PCs with the largest motions were selected and plotted for comparison. These PCs provide the main information about the spread of data points in the conformational space, indicating the global motion of the protein during simulations. To investigate the FEL, the gmx sham tool was employed to combine the reaction co-ordinates of the PCs with the most significant movements. The FEL plots were generated using MATLAB. All MD simulations and analyses were performed using high-performance computing resources at the University of Texas at Arlington, and through a generous startup allocation provided by the Extreme Science and Engineering Discovery Environment, which has since transitioned to the ACCESS (Advanced Cyberinfrastructure Coordination Ecosystem: Services & Support) program.

### Cloning, protein expression, and protein purification

The cloning, expression, and purification for WT caspase-3 were carried out as previously described [14,63]. Procaspase-3 and procaspase-8 network mutants (23 mutations) and other caspase-8 point mutants (caspase-8 H304A, caspase-8 K351A + K353A, and caspase-8 F310A + F340A + F355A + F399A) were all cloned into pET21b + plasmid, and sequences were confirmed by Sanger sequencing. Proteins were expressed in *E. coli* Lemo21(DE3) cells from NEB. We used constructs of caspase-8 where the pro-domain was removed to compare results with our previous studies [15]. Protein expression was carried out as previously described for procaspase-3, with the exception that we induced expression using 0.5 mM IPTG as the final concentration at 20°C based on optimizing expression studies [63]. After 16 h, cells were harvested by centrifugation at 5,000 rpm for 15 min, washed, and resuspended in buffer A (100 mM Tris-HCl, 100 mM NaCl, pH 7.5). The cells were lysed using a French press, followed by centrifugation of lysate at 15,000 rpm to separate supernatant from cell debris. All the mutants appeared in the pellet; hence, we performed denaturing purification by homogenizing the pellet in buffer B (6M urea, 100 mM Tris-HCl, 100 mM NaCl, pH 7.5) overnight and then by centrifuging at 15,000 rpm to release the proteins from inclusion bodies. The supernatant was then incubated on His-bind resin for 1 h, washed by ten column volumes of buffer B, followed by gradient elution of imidazole from 0 mM to 500 mM in buffer B. The samples were analyzed by SDS page, and pure samples were collected. The samples were dialyzed and refolded overnight at 4°C in buffer A containing 1 mM DTT and were then subjected to ion exchange chromatography (DEAE-sepharose) to separate impurities and further purify samples as previously described, with the exception that we used NaCl gradient instead of KCl gradient [63]. The samples were again dialyzed overnight at 4°C in buffer A containing 1 mM DTT, concentrated, and stored at −80°C.

### Folding studies preparation and data collection

Proteins were thawed on ice, and subsequent folding and unfolding experiments were conducted in accordance with previously established protocols [64]. For unfolding and refolding studies, protein samples were prepared in phosphate buffer (pH 7.5, 1 mM DTT) with urea concentrations ranging from 0 to 9 M. Sample preparations and data collection using fluorescence emission spectroscopy and circular dichroism spectropolarimetry for unfolding and refolding studies were carried out as described before [15].

### Analysis and global fitting of data for equilibrium folding/unfolding

Unfolding and refolding data from fluorescence emission and circular dichroism studies were fit globally to the appropriate model as described previously [15,64]. Briefly, we had a total of ten datasets comprising folding data at 2 μM, 6 μM, and 8 μM protein concentrations for each of the mutants. As there was no protein concentration dependence, the data were best fit to a monomeric three-state equilibrium folding model for caspase-3, caspase-8, and the caspase network mutants (23 mutants), as the data show a change in slope at ~4M urea. In the three-state monomeric model, the native conformation (N) unfolds to a partially folded intermediate conformation (I) before fully unfolding (U).


(eqn. 1) $$N\underset{K_{1}}{↔}I\underset{K_{2}}{↔}U$$

The data for the allosteric pocket mutants displayed a single transition and were best fit to a two-state folding model (Figure 6).


(eqn. 2) $$N\underset{K_{1}}{↔}U$$

Detailed descriptions of both folding models have been previously documented [64]. Global fitting of data to the models described in the equations above was done utilizing Igor Pro (WaveMetrics, Inc.). The global fitting is depicted as solid lines in . The thermodynamic parameters, ΔG^°^ and m-values, from the global fits are presented in Supplementary Table S1.

## Supplementary material

Online supplementary material 1

Online supplementary material 2

Online supplementary video 1

Online supplementary video 2

Online supplementary video 3

Online supplementary video 4

Online supplementary video 5

Online supplementary video 6

## Data Availability

All data are contained in the article and supporting information.

## Open Access

This article has been published open access under our Subscribe to Open programme, made possible through the support of our subscribing institutions, learn more here: https://portlandpress.com/pages/open_access_options_and_prices#conditional

## Abbreviations

AOA: ancestor of all
AOE: ancestor of all effectors
AOI: ancestral sequences of all initiators
APR: ancestral protein reconstruction
Anc6: ancestor of caspase-6
Anc3/7: ancestor of caspase-3/-7
BC: betweenness centrality
DC: degree centrality
DEAE: diethylaminoethyl
FEL: free energy landscape
IPTG: isopropyl β-D-1-thiogalactopyranoside
21M: 21 mutations
MD: molecular dynamics
NEB: New England Biolabs
PCA: principal component analysis
PCs: principal components
PDB: protein data bank
SDS: sodium dodecyl sulfate
WT: wildtype
cFLIP: cellular FLICE-like inhibitory protein

## References

[1] Ingles-Prieto A. Ibarra-Molero B. Delgado-Delgado A. Perez-Jimenez R. Fernandez J.M. Gaucher E.A et al 2013 Conservation of protein structure over four billion years Structure 21 1690 1697 10.1016/j.str.2013.06.020 23932589 PMC3774310

[2] Ortlund E.A. Bridgham J.T. Redinbo M.R. Thornton J.W 2007 Crystal structure of an ancient protein: evolution by conformational epistasis Science 317 1544 1548 10.1126/science.1142819 17702911 PMC2519897

[3] Campbell E. Kaltenbach M. Correy G.J. Carr P.D. Porebski B.T. Livingstone E.K. et al 2016 The role of protein dynamics in the evolution of new enzyme function Nat. Chem. Biol. 12 944 950 10.1038/nchembio.2175 27618189

[4] Worth C.L. Gong S. Blundell T.L 2009 Structural and functional constraints in the evolution of protein families Nat. Rev. Mol. Cell Biol. 10 709 720 10.1038/nrm2762 19756040

[5] Berger B. Daniels N.M. William Yu Y 2016 Computational biology in the 21st century: scaling with compressive algorithms Commun ACM, Association for Computing Machinery 59 72 10.1145/2957324 PMC5615407 28966343

[6] Wu K. Karapetyan E. Schloss J. Vadgama J. Wu Y 2023 Advancements in small molecule drug design: A structural perspective Drug Discov. Today 28 103730 10.1016/j.drudis.2023.103730 37536390 PMC10543554

[7] Clark A.C 2016 Caspase allostery and conformational selection Chem. Rev. 116 6666 6706 10.1021/acs.chemrev.5b00540 26750439

[8] MacKenzie S.H. Clark A.C 2012 Death by caspase dimerization Adv. Exp. Med. Biol. 747 55 73 10.1007/978-1-4614-3229-6_4 22949111 PMC3877935

[9] Joglekar I. Clark A.C 2023 Sequential unfolding mechanisms of monomeric caspases Biochemistry 62 1878 1889 10.1021/acs.biochem.3c00004 37337671 PMC10286309

[10] Boatright K.M. Renatus M. Scott F.L. Sperandio S. Shin H. Pedersen I.M. et al 2003 A unified model for apical caspase activation Mol. Cell 11 529 541 10.1016/S1097-2765(03)00051-0 12620239

[11] Sakamaki K. Satou Y 2009 Caspases: evolutionary aspects of their functions in vertebrates J. Fish Biol. 74 727 753 10.1111/j.1095-8649.2009.02184.x 20735596 PMC2779465

[12] Salvesen G.S. Dixit V.M 1999 Caspase activation: the induced-proximity model Proc. Natl. Acad. Sci. USA 96 10964 10967 10.1073/pnas.96.20.10964 10500109 PMC34227

[13] Shi Y 2004 Caspase activation: revisiting the induced proximity model Cell 117 855 858 10.1016/j.cell.2004.06.007 15210107

[14] Bose K. Clark A.C 2001 Dimeric procaspase-3 unfolds via a four-state equilibrium process Biochemistry 40 14236 14242 10.1021/bi0110387 11714277

[15] Nag M. Clark A.C 2023 Conserved folding landscape of monomeric initiator caspases J. Biol. Chem. 299 103075 10.1016/j.jbc.2023.103075 36858199 PMC10074801

[16] Shrestha S. Clark A.C 2021 Evolution of the folding landscape of effector caspases J. Biol. Chem. 297 101249 101249 10.1016/j.jbc.2021.101249 34592312 PMC8628267

[17] MacKenzie S.H. Schipper J.L. England E.J. Thomas M.E. 3rd Blackburn K. Swartz P. et al 2013 Lengthening the intersubunit linker of procaspase 3 leads to constitutive activation Biochemistry 52 6219 6231 10.1021/bi400793s 23941397

[18] Zamaraev A.V. Kopeina G.S. Prokhorova E.A. Zhivotovsky B. Lavrik I.N 2017 Post-translational modification of caspases: the other side of apoptosis regulation Trends Cell Biol. 27 322 339 10.1016/j.tcb.2017.01.003 28188028

[19] Thomas M.E. Grinshpon R. Swartz P. Clark A.C 2018 Modifications to a common phosphorylation network provide individualized control in caspases Journal of Biological Chemistry 293 5447 5461 10.1074/jbc.RA117.000728 29414778 PMC5900778

[20] Li X. Wen W. Liu K. Zhu F. Malakhova M. Peng C. et al 2011 Phosphorylation of Caspase-7 by p21-activated Protein Kinase (PAK) 2 Inhibits Chemotherapeutic Drug-induced Apoptosis of Breast Cancer Cell Lines Journal of Biological Chemistry 286 22291 22299 10.1074/jbc.M111.236596 21555521 PMC3121375

[21] Alvarado-Kristensson M. Melander F. Leandersson K. Rönnstrand L. Wernstedt C. Andersson T 2004 p38-MAPK signals survival by phosphorylation of caspase-8 and caspase-3 in human neutrophils J. Exp. Med. 199 449 458 10.1084/jem.20031771 14970175 PMC2211830

[22] Matthess Y. Raab M. Knecht R. Becker S. Strebhardt K 2014 Sequential Cdk1 and Plk1 phosphorylation of caspase‐8 triggers apoptotic cell death during mitosis Mol. Oncol. 8 596 608 10.1016/j.molonc.2013.12.013 24484936 PMC5528627

[23] Gonzalvez F. Lawrence D. Yang B. Yee S. Pitti R. Marsters S et al 2012 TRAF2 Sets a threshold for extrinsic apoptosis by tagging caspase-8 with a ubiquitin shutoff timer Mol. Cell 48 888 899 10.1016/j.molcel.2012.09.031 23142077

[24] Tang Y. Joo D. Liu G. Tu H. You J. Jin J. et al 2018 Linear ubiquitination of cFLIP induced by LUBAC contributes to TNFα-induced apoptosis Journal of Biological Chemistry 293 20062 20072 10.1074/jbc.RA118.005449 30361438 PMC6311529

[25] Velázquez-Delgado E.M. Hardy J.A 2012 Zinc-mediated allosteric Inhibition of Caspase-6 Journal of Biological Chemistry 287 36000 36011 10.1074/jbc.M112.397752 22891250 PMC3476268

[26] Schneider M. Antes I 2022 SenseNet, a tool for analysis of protein structure networks obtained from molecular dynamics simulations PLoS ONE 17 e0265194 10.1371/journal.pone.0265194 35298511 PMC8929561

[27] Amadei A. Linssen A.B.M. Berendsen H.J.C 1993 Essential dynamics of proteins Proteins 17 412 425 10.1002/prot.340170408 8108382

[28] Grinshpon R.D. Shrestha S. Titus-McQuillan J. Hamilton P.T. Swartz P.D. Clark A.C 2019 Resurrection of ancestral effector caspases identifies novel networks for evolution of substrate specificity Biochemical Journal 476 3475 3492 10.1042/BCJ20190625 31675069 PMC6874516

[29] Keller N. Mares J. Zerbe O. Grütter M.G 2009 Structural and biochemical studies on procaspase-8: new insights on initiator caspase activation Structure 17 438 448 10.1016/j.str.2008.12.019 19278658

[30] Kannan N. Vishveshwara S 1999 Identification of side-chain clusters in protein structures by a graph spectral method 1 1Edited by J. M. Thornton J. Mol. Biol. 292 441 464 10.1006/jmbi.1999.3058 10493887

[31] Venkat A. Tehrani D. Taujale R. Yeung W. Gravel N. Moremen K.W. et al 2022 Modularity of the hydrophobic core and evolution of functional diversity in fold a glycosyltransferases J. Biol. Chem. 298 102212 102212 10.1016/j.jbc.2022.102212 35780833 PMC9364030

[32] Foutch D. Pham B. Shen T 2021 Protein conformational switch discerned via network centrality properties Comput. Struct. Biotechnol. J. 19 3599 3608 10.1016/j.csbj.2021.06.004 34257839 PMC8246261

[33] Bromberg S. Dill K.A 1994 Side-Chain Entropy and Packing in Proteins Protein Science, Cambridge University Press 10.1002/pro.5560030702 PMC2142898 7920265

[34] Fokas A.S. Cole D.J. Ahnert S.E. Chin A.W 2016 residue geometry networks: a rigidity-based approach to the amino acid network and evolutionary rate analysis Sci. Rep. 6 33213 10.1038/srep33213 27623708 PMC5021933

[35] Ashkenazy H. Abadi S. Martz E. Chay O. Mayrose I. Pupko T. et al 2016 ConSurf 2016: an improved methodology to estimate and visualize evolutionary conservation in macromolecules Nucleic Acids Res. 44 W344 50 10.1093/nar/gkw408 27166375 PMC4987940

[36] Yao L. Clark A.C 2022 Comparing the folding landscapes of evolutionarily divergent procaspase-3 Biosci. Rep. 42 1 13 10.1042/BSR20220119 PMC9208311 35670809

[37] Khan M.T. Ali S. Zeb M.T. Kaushik A.C. Malik S.I. Wei D.-Q 2020 Gibbs free energy calculation of mutation in PncA and RpsA associated with pyrazinamide resistance Front. Mol. Biosci. 7 52 10.3389/fmolb.2020.00052 32328498 PMC7160322

[38] David C.C. Jacobs D.J 2014 Principal component analysis: a method for determining the essential dynamics of proteins Methods Mol. Biol. 1084 193 226 10.1007/978-1-62703-658-0_11 24061923 PMC4676806

[39] Pisani P. Caporuscio F. Carlino L. Rastelli G 2016 Molecular dynamics simulations and classical multidimensional scaling unveil new metastable states in the conformational landscape of CDK2 PLoS ONE 11 e0154066 10.1371/journal.pone.0154066 27100206 PMC4839568

[40] Manrique P.D. Chakraborty S. Henderson R. Edwards R.J. Mansbach R. Nguyen K. et al 2023 Network analysis uncovers the communication structure of SARS-CoV-2 spike protein identifying sites for immunogen design iScience 26 105855 10.1016/j.isci.2022.105855 36590900 PMC9791713

[41] Yu H. Kim P.M. Sprecher E. Trifonov V. Gerstein M 2007 The importance of bottlenecks in protein networks: correlation with gene essentiality and expression dynamics PLoS Comput. Biol. 3 e59 10.1371/journal.pcbi.0030059 17447836 PMC1853125

[42] Foutch D. Pham B. Shen T 2021 Protein conformational switch discerned via network centrality properties Comput. Struct. Biotechnol. J. 19 3599 3608 10.1016/j.csbj.2021.06.004 34257839 PMC8246261

[43] Piana S. Sulpizi M. Rothlisberger U 2003 Structure-based thermodynamic analysis of caspases reveals key residues for dimerization and activity Biochemistry 42 8720 8728 10.1021/bi034032l 12873132

[44] Stetz G. Verkhivker G.M 2017 Computational analysis of residue interaction networks and coevolutionary relationships in the hsp70 chaperones: a community-hopping model of allosteric regulation and communication PLoS Comput. Biol. 13 e1005299 10.1371/journal.pcbi.1005299 28095400 PMC5240922

[45] Smith I.N. Thacker S. Seyfi M. Cheng F. Eng C 2019 Conformational dynamics and allosteric regulation landscapes of germline PTEN mutations associated with autism compared to those associated with Cancer Am. J. Hum. Genet 104 861 878 10.1016/j.ajhg.2019.03.009 31006514 PMC6506791

[46] Soundararajan V. Aravamudan M 2014 Global connectivity of hub residues in Oncoprotein structures encodes genetic factors dictating personalized drug response to targeted Cancer therapy Sci. Rep. 4 7294 10.1038/srep07294 25465236 PMC4252896

[47] Tazhigulov R.N. Gayvert J.R. Wei M. Bravaya K.B 2019 eMap: a web application for identifying and visualizing electron or hole hopping pathways in proteins J. Phys. Chem. B 123 6946 6951 10.1021/acs.jpcb.9b04816 31288524

[48] Záliš S. Heyda J. Šebesta F. Winkler J.R. Gray H.B. Vlček A 2021 Photoinduced hole hopping through tryptophans in proteins Proc. Natl. Acad. Sci. USA 118 10.1073/pnas.2024627118 PMC7980458 33836608

[49] Allison J.R 2020 Computational methods for exploring protein conformations Biochem. Soc. Trans. 48 1707 1724 10.1042/BST20200193 32756904 PMC7458412

[50] Grinshpon R.D. Williford A. Titus-McQuillan J. Clay Clark A 2018 The CaspBase: a curated database for evolutionary biochemical studies of caspase functional divergence and ancestral sequence inference Protein Sci. 27 1857 1870 10.1002/pro.3494 30076665 PMC6199153

[51] Waterhouse A.M. Procter J.B. Martin D.M.A. Clamp M. Barton G.J 2009 Jalview Version 2—a multiple sequence alignment editor and analysis workbench Bioinformatics 25 1189 1191 10.1093/bioinformatics/btp033 19151095 PMC2672624

[52] Pei J. Grishin N.V 2014 PROMALS3D: Multiple protein sequence alignment enhanced with evolutionary and three-dimensional structural information Methods in Molecular Biology, Humana Press Inc 1079 263 271 10.1007/978-1-62703-646-7_17/FIGURES/00171 PMC4506754 24170408

[53] Du J.-Q. Wu J. Zhang H.-J. Zhang Y.-H. Qiu B.-Y. Wu F. et al 2008 Isoquinoline-1,3,4-trione derivatives inactivate caspase-3 by generation of reactive oxygen species Journal of Biological Chemistry 283 30205 30215 10.1074/jbc.M803347200 18768468 PMC2662080

[54] Chai J. Wu Q. Shiozaki E. Srinivasula S.M. Alnemri E.S. Shi Y 2001 Crystal structure of a procaspase-7 zymogen: mechanisms of activation and substrate binding Cell, Cell 107 399 407 10.1016/S0092-8674(01)00544-X 11701129

[55] Vaidya S. Velázquez-Delgado E.M. Abbruzzese G. Hardy J.A 2011 Substrate-Induced conformational changes occur in all cleaved forms of caspase-6 J. Mol. Biol. 406 75 91 10.1016/j.jmb.2010.11.031 21111746 PMC3030624

[56] Wang Z. Watt W. Brooks N.A. Harris M.S. Urban J. Boatman D. et al 2010 Kinetic and structural characterization of caspase-3 and caspase-8 inhibition by a novel class of irreversible inhibitors Biochimica et Biophysica Acta (BBA) - Proteins and Proteomics 1804 1817 1831 10.1016/j.bbapap.2010.05.007 20580860

[57] Yu J.W. Jeffrey P.D. Shi Y 2009 Mechanism of procaspase-8 activation by c-FLIPL Proc Natl Acad Sci U S A, National Academy of Sciences 106 8169 8174 10.1073/pnas.0812453106 PMC2688887 19416807

[58] Thomsen N.D. Koerber J.T. Wells J.A 2013 Structural snapshots reveal distinct mechanisms of procaspase-3 and -7 activation Proc. Natl. Acad. Sci. USA 110 8477 8482 10.1073/pnas.1306759110 23650375 PMC3666719

[59] Murray J. Giannetti A.M. Steffek M. Gibbons P. Hearn B.R. Cohen F. et al 2014 Tailoring small molecules for an allosteric site on procaspase‐6 ChemMedChem 9 73 77 10.1002/cmdc.201300424 24259468

[60] Xu J.H. Eberhardt J. Hill-Payne B. González-Páez G.E. Castellón J.O. Cravatt B.F. et al 2020 Integrative X-ray structure and molecular modeling for the rationalization of procaspase-8 Inhibitor Potency and Selectivity ACS Chem. Biol. 15 575 586 10.1021/acschembio.0c00019 31927936 PMC7370820

[61] Shannon P. Markiel A. Ozier O. Baliga N.S. Wang J.T. Ramage D. et al 2003 Cytoscape: a software environment for integrated models of biomolecular interaction networks Genome Res. 13 2498 2504 10.1101/gr.1239303 14597658 PMC403769

[62] Meng S. Li Z. Ji Y. Ruff A.J. Liu L. Davari M.D. et al 2023 Introduction of aromatic amino acids in electron transfer pathways yielded improved catalytic performance of cytochrome P450s Chinese Journal of Catalysis 49 81 90 10.1016/S1872-2067(23)64445-6

[63] Pop C. Chen Y.R. Smith B. Bose K. Bobay B. Tripathy A. et al 2001 Removal of the pro-domain does not affect the conformation of the procaspase-3 dimer Biochemistry 40 14224 14235 10.1021/bi011037e 11714276

[64] Walters J. Milam S.L. Clark A.C 2009 Practical approaches to protein folding and assembly: spectroscopic strategies in thermodynamics and kinetics Meth. Enzymol. 455 1 39 10.1016/S0076-6879(08)04201-8 19289201 PMC2778058

